# Morphometric Analysis of Prostate Zonal Anatomy After Transurethral Resection of Prostate and Holmium Laser Enucleation of Prostate Using Magnetic Resonance Imaging: A Pilot Study

**DOI:** 10.5152/tud.2022.21326

**Published:** 2022-05-01

**Authors:** Abhishek Bhat, Jonathan E. Katz, Vedant K. Acharya, Khushi Shah, Ruben Blachman Braun, Nicholas Anthony Smith, R. Patricia Castillo, Hemendra N. Shah

**Affiliations:** 1 Department of Urology, Jackson Health System, Miami, FL; 2 Department of Urology, University of Miami Miller Faculty of Medicine, Miami, FL; 3 Department of Radiology, University of Miami Miller Faculty of Medicine, Miami, FL

**Keywords:** Transurethral resection of prostate, holmium laser enucleation of prostate, magnetic resonance imaging of prostate, prostate zonal anatomy, incidental prostate cancer, prostate-specific antigen

## Abstract

**Objective:**

The primary purpose was to compare the completeness of adenomectomy and zonal anatomy of prostate on magnetic resonance imaging prostate after transurethral resection of prostate and Holmium enucleation of prostate. The secondary purpose was to investigate the relationship between preoperative total prostate volume and postoperative transition zone and peripheral zone volume after both procedures.

**Material and methods:**

A retrospective review of all patients who underwent transurethral resection of prostate or Holmium enucleation of prostate over 3 years (2017-2020) and had at least 1 postoperative magnetic resonance imaging prostate was performed. Volume estimations of the prostate and individual zones were performed, and statistical comparisons were made to evaluate morphometric changes between the 2 procedures.

**Results:**

A total of 9 patients (mean age, 71.8 years) underwent transurethral resection of prostate and 12 patients (mean age, 66.9 years) underwent Holmium enucleation of prostate. The median pre-operative prostate volume in the Holmium enucleation of prostate group was higher than the transurethral resection of prostate group (101.5 g vs. 62 g; *P* = .102). However, there was a significant difference in the resected tissue weight favoring Holmium enucleation of prostate over transurethral resection of prostate (*P* value = .004). The postoperative transition zone and peripheral zone volume as calculated by magnetic resonance imaging remained relatively constant in both procedures. The peripheral zone volume on postoperative magnetic resonance imaging was found to be independent of transition zone volume even for very large-sized prostates.

**Conclusion:**

A well-performed transurethral resection of prostate or Holmium enucleation of prostate can nearly completely eliminate the transition zone volume, irrespective of the size of the prostate as confirmed by magnetic resonance imaging prostate. Additionally, the peripheral zone volume is consistent across the entire spectrum of the prostate size.

**Main Points**

- Debulking procedures for benign prostatic hyperplasia such as transurethral resection of the prostate and Holmium laser enucleation of prostate involve resection or enucleation of the transition zone (TZ).
- A well-performed bladder outlet procedure results in near-complete elimination of the TZ irrespective of the size of the prostate as confirmed by magnetic resonance imaging prostate
- The volume of peripheral zone is consistent across the entire spectrum of prostate size.

## Introduction

Benign prostatic hyperplasia (BPH) and lower urinary tract symptoms (LUTS) are known to affect 70% of US men 60-69 years of age and 80% of those 70 years of age or older.^1^ Despite widespread use of medical management for symptomatic prostate enlargement, a significant number of patients need surgical intervention. Transurethral resection of the prostate (TURP) continues to be considered the gold standard for the surgical management of BPH. Complete resection of all adenomatous tissue is recommended by most resectionists as the standard TURP technique.^2^ Holmium laser enucleation of prostate (HoLEP) allows complete enucleation of even large adenomas leaving behind only the peripheral zone (PZ).^3^ It is therefore recommended as a size-independent procedure for the treatment of an enlarged prostate by the American Urology Association.^4^

There is evidence to suggest that clinical improvement after TURP correlates significantly with the completeness of resection of the obstructing adenoma.^5^ In real life, the definition of completeness of adenomectomy after TURP and HoLEP is more subjective and depends widely on surgeon’s experience. Although there has been extensive literature comparing functional outcomes after TURP and HoLEP,^6^ there is no radiological evidence comparing completeness of adenomectomy between the techniques. Few randomized trials have measured the residual weight of prostates after these procedures. However, the individual contributions of the PZ and the transitional zone (TZ) to the total residual prostate (RP) tissue mass has not been studied.^7,8^ Hence, it is unclear if postoperative residual total prostate volume (TPV) represents only the PZ and anterior fibromuscular stroma or some residual TZ as well. In other words, magnetic resonance imaging (MRI) estimation of postoperative TPV has not been well studied as a tool to assess completeness of adenomectomy after these procedures. Improvements in prostate imaging with MRI have provided excellent insight into the visualization of the zonal anatomy of the prostate.^9^ The primary objective of our study was to compare the completeness of adenomectomy and zonal volumes of postoperative PZ and TZ with respect to the TPV after TURP and HoLEP. The secondary objective was to investigate the relationship between preoperative TPV and postoperative TZ and PZ volume after both the procedures. 

## Material and Methods

### Patients

This was an Institutional Research Board (IRB)-approved study (IRB Approval Number: 20180511). The IRB approval included Ethics Committee approval as well. Medical records of all patients who underwent either a TURP (group 1) or HoLEP (group 2) for the treatment of BPH at the study institution over 3 years between February 2017 and February 2020 were retrospectively reviewed to identify those patients who had at least 1 postoperative MRI prostate within a year of the procedure. After informed consent had been obtained from all included patients, all subjects with an elevated prostate-specific antigen (PSA) preoperatively were evaluated with MRI and/or 4K score, followed by prostate biopsy, when appropriate to rule out prostate cancer (PCa). Patients diagnosed with PCa before TURP or HoLEP were excluded from the present study. Postoperative MRIs were performed in these patients either as a part of active surveillance for incidentally diagnosed low-risk PCa on histopathological examination of resected prostate specimen after the procedures or for evaluation of persistently elevated PSA (>1 ng/mL) after HoLEP. All patients with Prostate Imaging Reporting and Data System (PIRADS) 4 or 5 lesions on postoperative MRI were excluded from the study as the size of these lesions might have influenced PZ volume. At present, although the PIRADS classification is not universally accepted post-debulking surgeries of the prostate, the institutional and departmental protocols do utilize the classification as the biopsy correlation results have been consistently accurate and there is no better radiological substitute available. 

The choice of the surgical procedure was arrived at after a process of informed decision-making between the operating surgeons and the patient. Preoperative prostate volume was estimated by ultrasound, computed tomography scan, or MRI in all except 2 patients in whom estimation was based on digital rectal examination. All HoLEP were performed by a single surgeon (HNS) with experience of > 2000 HoLEP/TURP surgeries, and TURPs were performed by 1 of the 3 surgeons with experience of >1000 TURPs with the aim of complete adenoma resection. The volume of resected tissue was noted by a single uro-pathologist. All patients had a postoperative PSA estimation at 3 months. 

The time interval between surgical procedure and first MRI was noted. Every MRI performed was independently reviewed by a single radiologist (RC). Other peri-operative data analyzed included patients’ age, BMI, PSA values, weight of resected tissue, and histopathological diagnosis. The data of patients from both groups were compared to look for differences in prostate zonal anatomy after each procedure. The impact of preoperative prostate size and resected prostate weight on postoperative TZ and PZ volume was studied. 

### Magnetic Resonance Imaging

All MRI examinations were performed on a 3.0 T MR scanner (Trio and Skyra, Siemens Healthineers, Erlangen, Germany or Discovery, General Electric, Milwaukee, Wis, USA). The patients were imaged in supine position with a phased-array body coil placed over the pelvis. An endorectal coil was not utilized. The standard prostate MRI protocol used at our institution includes a single shot turbo/fast spin-echo T2-weighted axial and coronal localizer images through the pelvis; turbo/fast spin-echo T2-weighted axial, coronal, and sagittal small field of view images without fat saturation through the prostate and seminal vesicles; turbo/fast spin-echo T2-weighted axial images with fat saturation through the pelvis; echo-planar axial diffusion-weighted images through the prostate and gradient-echo T1-weighted axial images without fat saturation through the pelvis. Dynamic contrast-enhanced axial T1 images without fat saturation are obtained through the pelvis after injecting 0.1 mm/kg of multi-hance at 2.0 cm^3^ per second followed by a 20 mL saline flush. Dynamic contrast-enhanced images are obtained every 9.4 seconds for 5 minutes following injection of contrast.

The transverse size was measured in the axial plane at the area of maximal diameter, from the inner margin of the external prostatic capsule and the longitudinal diameters were measured in the midsagittal plane. The craniocaudal dimension was measured by using coronal images. For study purposes, the anterior fibromuscular stroma was considered as part of PZ volume. Total prostate volume (TPV), transition zone volume (TZV), and PSA density (PSAD) were evaluated. Peripheral zone volume was evaluated by subtracting TZV from the TPV. The prostate volume was calculated on T2-weighted images according to the “Prolate Ellipsoid” formula: volume = AP × transverse × length × 0.52.^10^

### Statistical Analysis

Statistical analysis was performed with Statistical Package for the Social Sciences 24 software (IBM SPSS Corp.; Armonk, NY, USA). Continuous variables were presented as mean ± standard deviation or medians and interquartile ranges [25th-75th] in accordance with data distribution and were analyzed with the Welch, Mann–Whitney *U* test, or Wilcoxon test as required. Categorical variables were presented as absolute variables and frequencies and were analyzed with the chi-square test or Fisher exact test as required. A *P* value <.05 was considered statistically significant.

## Results

A total of 21 patients met the inclusion criteria for this pilot study. Nine patients underwent TURP and the remaining 12 patients underwent HoLEP. Patients’ demographic data including age, body mass index (BMI), and pre- and postprocedural PSA levels were comparable between both groups (Table 1). Although the median preoperative prostate volume in the HoLEP group was higher than the TURP group, the difference was not found to be statistically significant (101.5 g vs. 62 g; *P* = .102), and the weight of resected prostate in HoLEP was significantly greater than TURP (63.2 g in HoLEP vs. 15 g in TURP, *P* value = .004). 

Preprocedure prostate biopsy was performed in 8/9 patients in the TURP group versus 4/12 patients in the HoLEP group and was negative for PCa. However, more than 75% of the patients in both groups (9/9 in the TURP group and 8/12 in the HoLEP group) were diagnosed to have varying grades of incidental PCa on postoperative histopathological evaluation of prostate tissue. Majority of patients were diagnosed with low-risk (Gleason Grade group 1) PCa. All patients with Gleason Grade group 1 continued to be on active surveillance, whereas men with intermediate-risk and high-risk PCa have been treated with either radiation therapy or radical prostatectomy.

**Figure 1:**
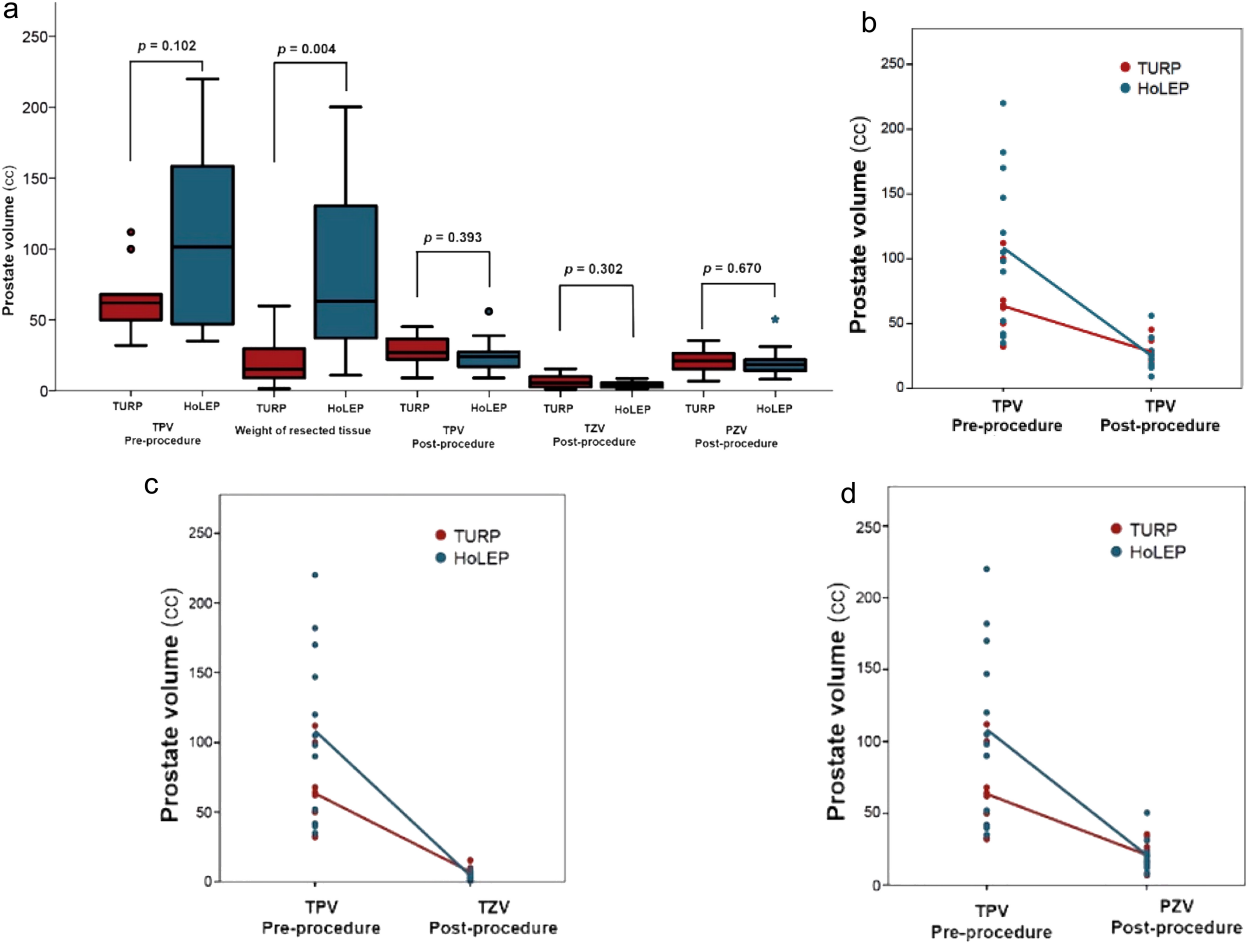
a-d. Comparison between zonal anatomy post-TURP and HoLEP. TURP, Transurethral resection of the prostate; HOLEP, Holmium laser enucleation of prostate.

**Figure 2:**
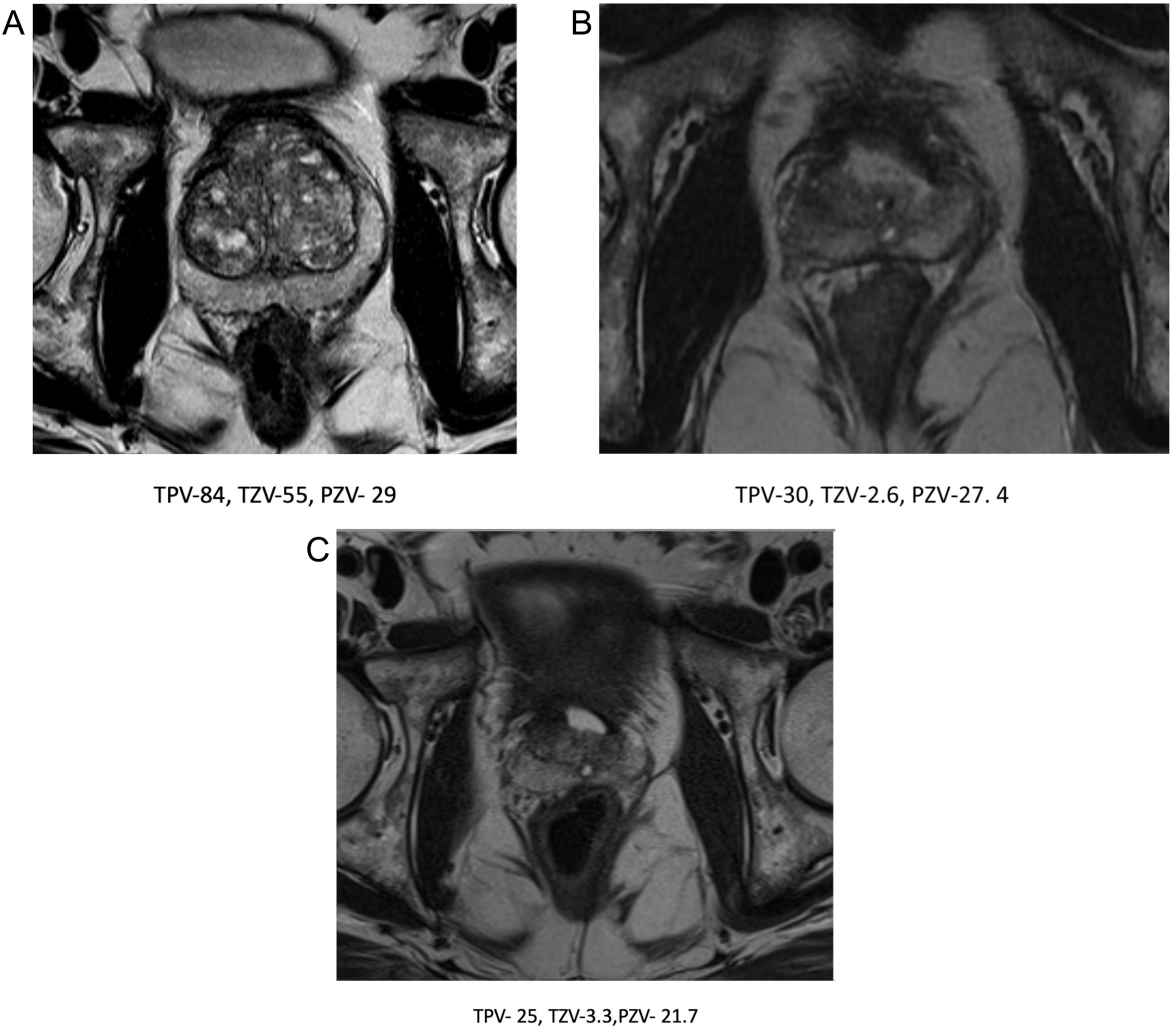
a-c. T2 weighted axial MRI images before and 6- week and 3 year after TURP procedure (volume in cc).

**Figure 3:**
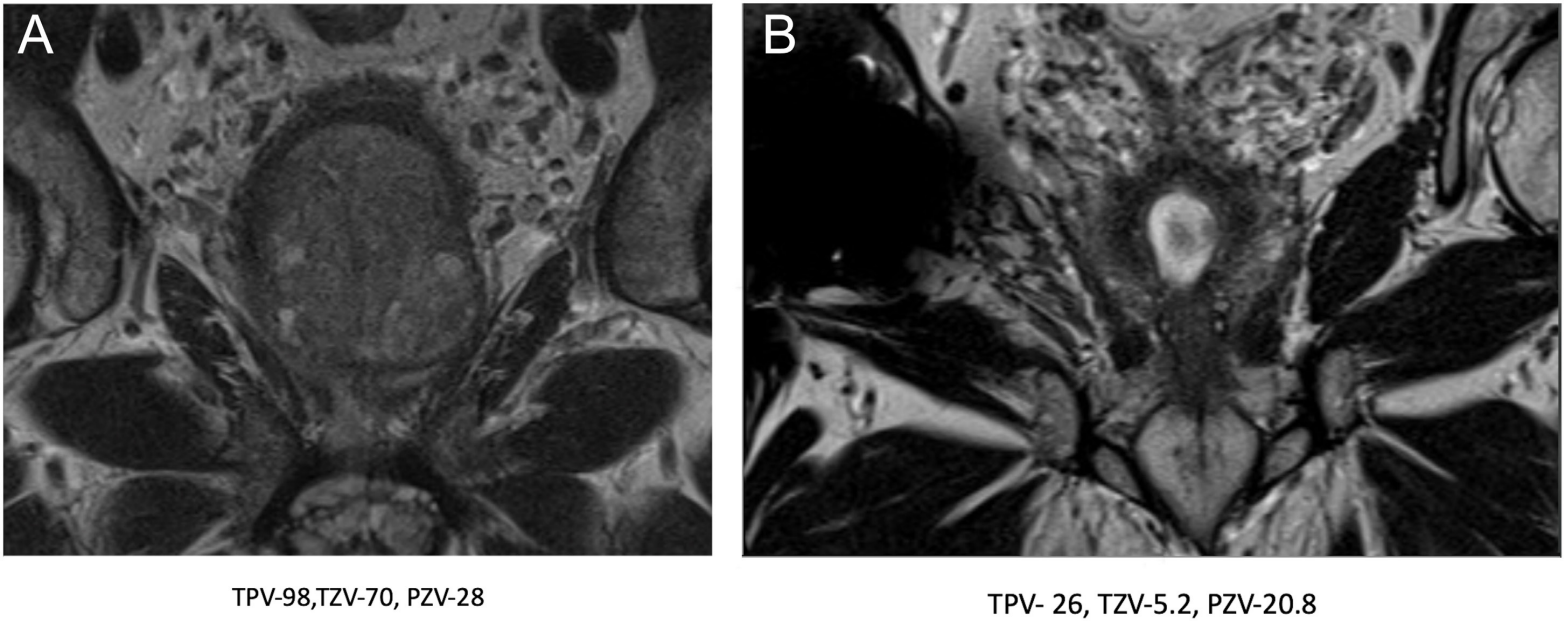
a,b. T2 weighted coronal MRI images before and 7 month after HoLEP procedure (volume in cc).

**Figure 4:**
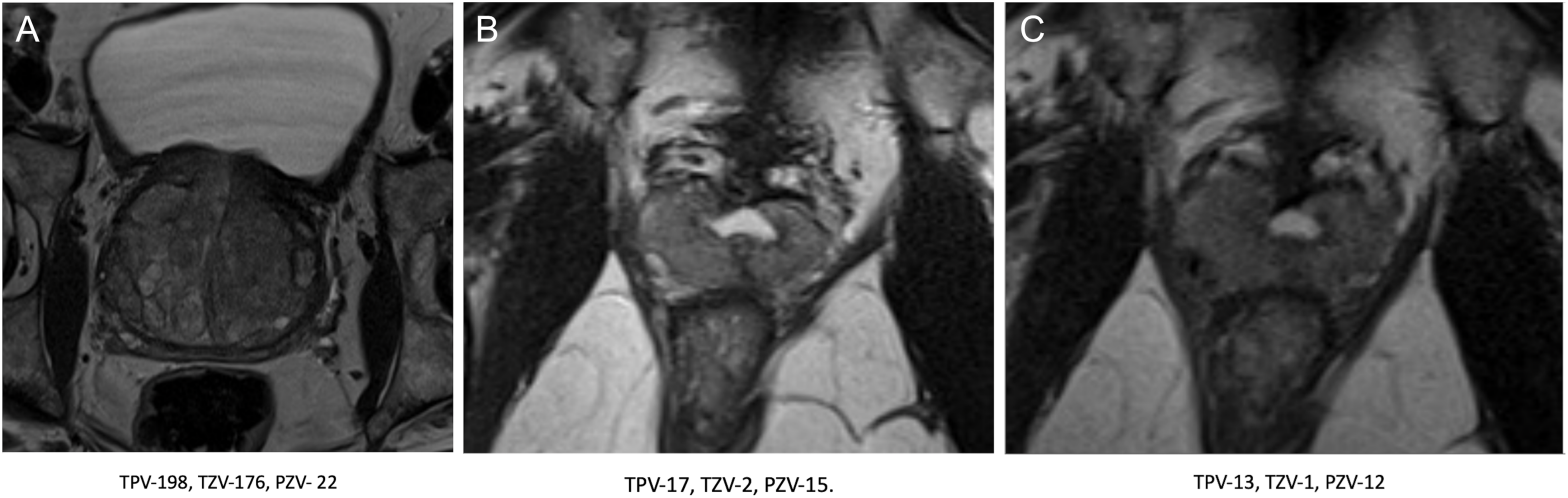
a-c. T-2 weighted images of prostate (axial view) before and 4 month and 1.5 year after HoLEP procedure (volume in cc).

**Figure 5:**
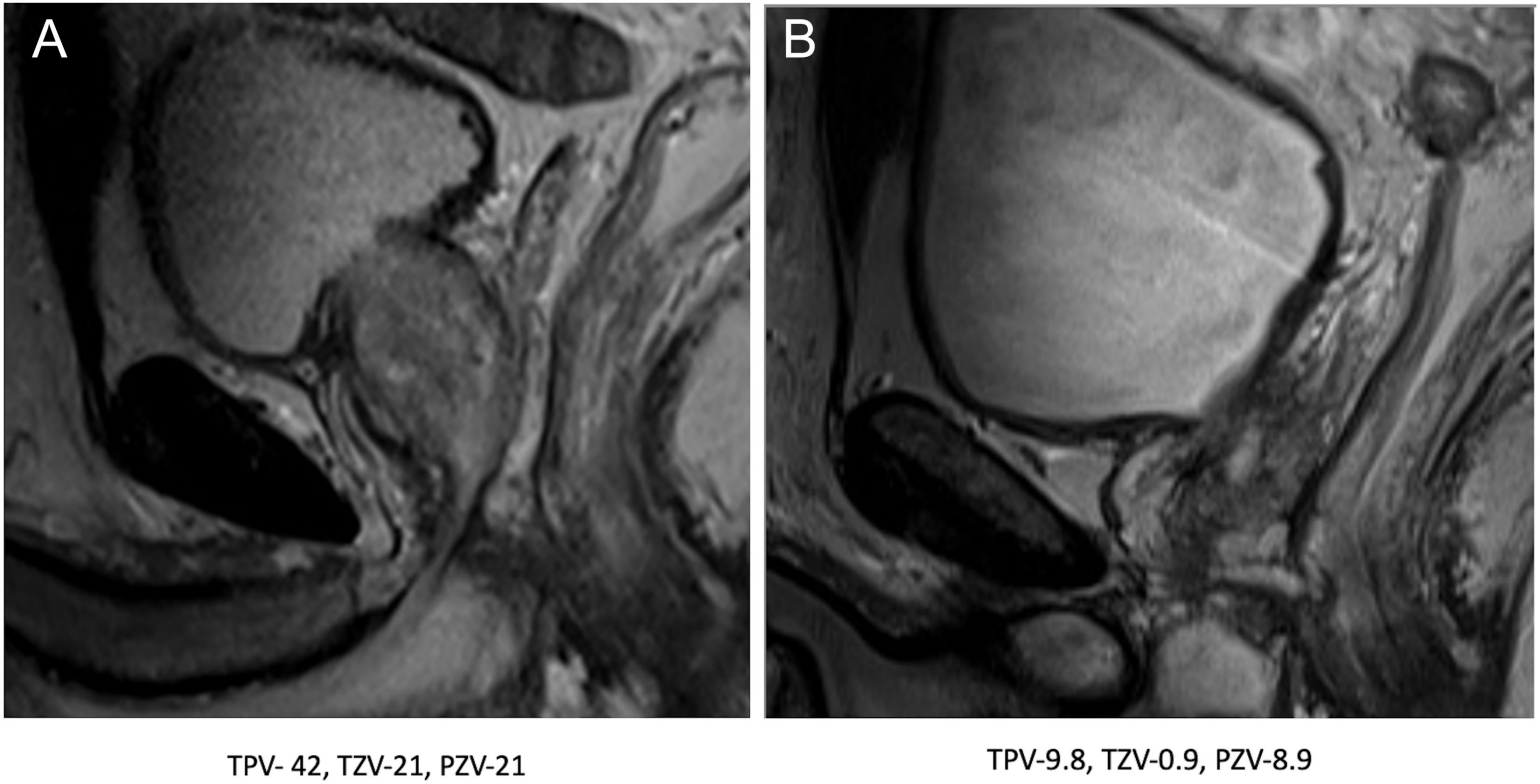
a,b. T-2 weighted images of prostate (coronal view) before and 2 month after HoLEP procedure (volume in cc).

The median postprocedure TPV was approximately the same in both groups (24.1 g in HoLEP group vs. 27 g in TURP group, *P* = .393). In terms of the prostate morphology on the MRI, there was no significant difference between TPV, the PZV, and therefore the TZ Index (TZI) which was 0.22 in group 1 and 0.20 in group 2 (Figure 1A). The difference in postoperative PSAD was also found to be statistically insignificant (*P* value = .522) (Table 1). The postoperative TZV and PZV remained relatively constant irrespective of preoperative prostate size. The median PZV on postoperative MRI ranged between 11.8 and 22.3 cm^3^ and was found to be independent of preoperative TPV (Figure 1b, 1c, 1d). The demonstration of this can be seen in 3 patients of varying prostate sizes treated with HoLEP wherein the entire TZ can be seen to be enucleated leaving behind a similar-sized PZ in prostates of all sizes (Figures 2A, 2B, 2C, 3A, 3B, 4A, 4B, 4C). Additionally, the comparison of pre- and postprocedure MRI in selected patients from our study provides an anatomical proof of concept that “a complete removal of prostate adenoma” by any surgical technique results in complete removal of TZ thereby leaving behind PZ of prostate along with a widely open prostatic urethra (Figures 2A, 2B, 2C, 3A, 3B, 4A, 4B, 4C, 5A, 5B).

## Discussion

Hastak et al^11^ initially reported the morphological changes in the prostate on transrectal ultrasound (TRUS) after 6-10 weeks post-TURP. Subsequent studies confirmed that the TPV measured by TRUS is comparable with findings on multiparametric MRI and both have excellent agreement with real prostate weight of the surgical specimens.^12,13^ However, TZV measurement on TRUS is operator dependent and its accuracy is controversial.^14^ Some authors report significant discrepancies between TRUS determination of TZV when compared with enucleated adenoma weight.^15^

In our pilot study, we evaluated the RP morphology after TURP and HoLEP by multiparametric MRI. Although the median preprocedure prostate volume was almost double in the HoLEP group as compared to the TURP group and the resected prostate in HoLEP was significantly greater than TURP, the median postprocedure TPV was approximately the same in both groups. The resected volume variation between HoLEP and TURP in our series was similar to other contemporary studies which also demonstrated increased resected tissue volume with HoLEP.^16^ The median resected weight was only 15 g in TURP group when the median pre-op volume of 62 g. This might indicate incomplete resection, though postoperative MRI PZV is equivalent between both groups suggesting complete resection of TZ in both groups. The median difference between preoperative prostate volume and the combined resected tissue volume and residual tissue volume on MRI is approximately 20 cm^3^ for TURP group and 14.2 cm^3^ in HoLEP group. These findings are difficult to explain. It can be hypothesized that more tissue might be lost during vaporization during bipolar TURP as compared to HoLEP.

The use of post-TURP MRI was earlier described by Abt et al^17^ who conducted a randomized control trial (RCT) comparing the outcome of prostate artery embolization with TURP.^17^ Fifty-one patients in the study, with a mean pre-operative prostate volume of 56.5 ± 31.1 cm^3^ on MRI underwent TURP. A postoperative MRI performed on these patients at an interval of 12 weeks revealed an RP volume of 27.16 cm^3^. Although these authors did not mention TZ and PZ volume of RP, their findings of TPV after TURP are similar to findings noted in our study. Conversely, in an RCT comparing HoLEP with TURP for the treatment of prostates 40-200 g, mean prostate volume on TRUS reduced at 6 months from 77.8 ± 5.6 g (42-152 g) to 28.4 ± 1.8 g (13-43 g) after HoLEP and 85.8 ± 5.4 g (46-156 g) to 46.6 ± 4.4 g (26-96 g) after TURP. The higher residual TPV in TURP groups probably indicates the incomplete adenomectomy of these larger prostates. At 7 years follow-up, it was noted that although none of the patients initially enrolled for HoLEP required surgery for prostate regrowth, 3 patients initially assigned to TURP arm had symptomatic prostate regrowth needing subsequent HoLEP.^18^ This indicates that complete adenomectomy translates into durable long-term outcomes. The median prostate size in TURP group in our series as well as from series published by Abt et al^17^ was smaller than that in the Gilling study [mean 85.8 ± 5.4 (46-156) g] thereby possibly enabling complete resection of adenoma to the level of the capsule.^18^ We noted that the median TZV was 4.5 cm^3^ in the HoLEP group and 5.8 cm^3^ in the TURP group, further confirming near-complete adenomectomy in both groups in our study. The completeness of adenomectomy after endoscopic enucleation is also independent of the energy source used. In an RCT comparing Thulium Laser Vapoenucleation of the prostate (ThuVEP) versus HoLEP, the median prostate size on TRUS reduced at 1-month follow-up from 82.5 g (47.75-100 g) to 20 gm (11.75-30 g) after ThuVEP and 77.5 g (45.75-110.25 g) to 16 g (11-27.5 g) after HoLEP.^8^

Interestingly, independent of whether patients underwent HoLEP or TURP, median PZV was found to be similar in both groups (18.5 g in HoLEP group vs. 21.2 g in TURP group). This corroborates results from a study by Meikle et al^19^ who noted that both TZ and PZ of the prostate grow with age, but once the TPV exceeds 30 g, the size of the PZ becomes attenuated. There is further evidence that the PZV is independent of TPV even for very large-sized prostates as in our study (Figure 1B, 1C, 1D). 

We employed “Prolate Ellipsoid” formula for calculating prostate volume.^20^ The same method was used to calculate the volume of the TZ before and after the procedure.^10,21,22^ We did not use the “Bullet formula” (L × H × W × 5π/24) or (L × H × W × 0.65) which has been proposed by some as a potentially superior formula in prostate volume estimation.^23^ Conflicting evidence exists regarding which formula is superior. Lee et al^20^ concluded that the prolate ellipsoid formula is accurate in estimating prostate volume using either TRUS or MRI. However, another study concluded that the ellipsoid formula consistently underestimates prostate size by 10% almost 80% of the time.^24^ This consistent volume underestimation when using the ellipsoid formula was also reported in a different study.^25^ The Prolate Ellipsoid formula also has the limitation of measuring the empty fossa volume and we overcame this by correlating findings with volumetric assessment by DynaCAD Prostate MR image analysis system®.

The clinical results after TURP were shown to correlate significantly with completeness of resection of the obstructing adenoma. In a study by Chen et al^5^ wherein TRUS was performed on 40 men at 16 weeks after TURP, the weight of RP was noted to be 22.6 ± 13.1 g. It was also noted that the RP weight ratio at 16 weeks provided a good estimate of clinical results, with better clinical outcome associated with a smaller RP weight ratio. The completeness of adenoma removal also directly correlates with the durability of clinical outcome.^18^ We hypothesized that after complete adenomectomy, the residual PZ remains the only source of PSA. Since the volume of PZ remains constant in all patients irrespective of the preoperative total prostate volume, we noted similar PSAD among both groups, and difference in postoperative PSAD was statistically insignificant (*P*-value = .522). 

The limitations of our study include the relatively small numbers of patients making it difficult to reach statistical significance, but it must be emphasized that postprocedural MRI is not a standard clinical practice and the fact that these are results of an initial pilot study which are being carried forward as an elaborate clinical trial. We also did not correlate postprocedure prostate morphology and clinical outcome. It will be interesting to prospectively evaluate the impact of postprocedure RP volume on degree and durability of clinical improvement after procedures for BPH. Detection of preoperative prostate volumes of patients by USG, CT, or MRI may cause heterogeneity in standardization. Two patients had volumetric assessment preoperatively with DRE; however, they had postoperative MRI volume estimation and were therefore included in the study. It would also have been preferable to have comparable preoperative prostate volume in both groups. Lastly, there are different formulae available for prostate size estimation, and although there may be recent evidence in favor of the Bullet formula, there is ample evidence to support the use of prolate ellipsoid formula as well. Despite these shortcomings, to the best of our knowledge, our study is the first to report and compare TZV after HoLEP and TURP and provide anatomical evidence regarding the completeness of adenomectomy after both procedures.

Based on the findings of our study and those from the literature, we propose that after a complete adenomectomy the RP measurement should be 20 ± 5 g. Patients with higher-than-expected RP volume should be an indication of incomplete adenomectomy and should be counseled appropriately about the possibility of relapse of their urinary symptoms in long term. The option of choosing the appropriate procedure should rest with the operating surgeon and should be based on his expertise.

In conclusion, bladder outlet procedures for BPH, whether TURP or HoLEP result in near-complete elimination of the TZ, irrespective of the preoperative size of the prostate as confirmed by MRI prostate. Additionally, the postprocedural PZ volume on MRI prostate remains remarkably constant and independent of preoperative prostate size. Further higher-impact prospective studies and trials are the need of the hour to validate the impact of completeness of resection on durability of outcomes. 

## Figures and Tables

**Table 1.** Comparison of the Perioperative Variables Between Patients Who Underwent TURP Versus HoLEP

Variables	TURP n = 9 (42.9%)	HoLEP n = 12 (57.1%)	*P*
Age (year)	71.8 ± 11.2	66.9 ± 7.7	.281^a^
BMI (kg/m^2^)	28.3 ± 5.4	28.9 ± 5.6	.779^a^
Preprocedure prostate volume (cm^3^)	62 [41.5-84]	101.5 [44.5-164.3]	.102^b^
Preprocedure PSA (ng/mL)	3.2 [1.3-5.3]	3.9 [2.8-7.1]	.434^b^
Preoperative biopsy			
Yes (%)	8 (88.9%)	4 (33.3%)	
No (%)	1 (11.1%)	8 (66.7%)	.024^d^
Weight of resected tissue (g)	15 [5.8-30.5]	63.2 [35.8-131.5]	.004^b^
Postoperative PSA (ng/mL)	1.1 [0.9-2.5]	0.7 [0.4-1.2]	.270^b^
Biopsy diagnosis after procedure			
BPH (%)	0	2 (16.7%)	
Gleason Grade group 1 (%)	5 (55.6%)	6 (50%)	
Gleason Grade group 2 (%)	2 (22.2%)	2 (16.7%)	
Gleason Grade group 3 (%)	1 (11.1%)	0	
Gleason Grade group 4 (%)	0	1 (8.3%)	
Gleason Grade group 5 (%)	1 (11.1%)	1 (8.3%)	.588^c^
Time interval between procedure and MRI (day)	160 [74-335]	121 [75.8-26.8]	.644^b^
Postprocedure TPV (cm^3^)	27 [21.9-38]	24.1 [16.7-28.3]	.393^b^
Postprocedure TZV (cm^3^)	5.80 [2.60-12.65]	4.50 [2.15-5.75]	.302^b^
Postprocedure PZV (cm^3^)	21.2 [11.8-30.3]	18.5 [13.9-22.3]	.670^b^
Postprocedure TZ index	0.22 [0.09-0.36]	0.20 [0.10-0.24]	.592^b^
Postprocedure PSA density (ng/mL)	0.051 [0.028-0.097]	0.036 [0.020-0.088]	.522^b^

BPH, benign prostatic hyperplasia; BMI, body mass index; MRI, magnetic resonance imaging; PSA, prostate-specific antigen; TPV, total prostate volume; PZV, peripheral zone volume; TZV, transition zone volume. Mean ± standard deviation; medians and interquartile ranges [25th-75th].

^a^ Welch test, ^b^ Mann–Whitney *U* test; ^c^Chi-square, ^d^ Fisher exact test.

## References

[1] Wei JT Calhoun E Jacobsen SJ . Urologic diseases in America project: benign prostatic hyperplasia. J Urol. 2005;173(4):1256 1261. 10.1097/01.ju.0000155709.37840.fe) 15758764

[2] Reich O Gratzke C Bachmann A et al. Morbidity, mortality and early outcome of transurethral resection of the prostate: a prospective multicenter evaluation of 10,654 patients. J Urol. 2008;180(1):246 249. 10.1016/j.juro.2008.03.058) 18499179

[3] Oh SJ Current surgical techniques of enucleation in holmium laser enucleation of the prostate. Investig Clin Urol. 2019;60(5):333 342. 10.4111/icu.2019.60.5.333) PMC6722407 31501795

[4] Foster HE Dahm P Kohler TS et al. Surgical management of lower urinary tract symptoms attributed to benign prostatic hyperplasia: AUA guideline Amendment 2019. J Urol. 2019;202(3):592 598. 10.1097/JU.0000000000000319) 31059668

[5] Chen SS Hong JG Hsiao YJ Chang LS . The correlation between clinical outcome and residual prostatic weight ratio after transurethral resection of the prostate for benign prostatic hyperplasia. BJU Int. 2000;85(1):79 82. 10.1046/j.1464-410x.2000.00433.x) 10619951

[6] Zhong J Feng Z Peng Y Liang H . A systematic review and meta-analysis of efficacy and safety following holmium laser enucleation of prostate and transurethral resection of prostate for benign prostatic hyperplasia. Urology. 2019;131:14 20. 10.1016/j.urology.2019.03.034) 31129190

[7] Tan AHH Gilling PJ Kennett KM Frampton C Westenberg AM Fraundorfer MR . A randomized trial comparing holmium laser enucleation of the prostate with transurethral resection of the prostate for the treatment of bladder outlet obstruction secondary to benign prostatic hyperplasia in large glands (40 to 200 grams). J Urol. 2003;170(4 Pt 1):1270 1274. 10.1097/01.ju.0000086948.55973.00) 14501739

[8] Becker B Herrmann TRW Gross AJ Netsch C . Thulium vapoenucleation of the prostate versus holmium laser enucleation of the prostate for the treatment of large volume prostates: preliminary 6-month safety and efficacy results of a prospective randomized trial. World J Urol. 2018;36(10):1663 1671. 10.1007/s00345-018-2321-8) 29730838

[9] Sklinda K Frączek M Mruk B Walecki J . Normal 3T MR anatomy of the prostate gland and surrounding structures. Adv Med. 2019;2019:3040859. 10.1155/2019/3040859) 31276002 PMC6558623

[10] Turkbey B Rosenkrantz AB Haider MA et al. Prostate Imaging Reporting and Data System Version 2.1: 2019 update of Prostate Imaging Reporting and Data System Version 2. Eur Urol. 2019;76(3):340 351. 10.1016/j.eururo.2019.02.033) 30898406

[11] Hastak SM Gammelgaard J Holm HH . Transrectal ultrasonic volume determination of the prostate--a preoperative and postoperative study. J Urol. 1982;127(6):1115 1118. 10.1016/s0022-5347(17)54258-5) 6177876

[12] Tewari A Indudhara R Shinohara K et al. Comparison of transrectal ultrasound prostatic volume estimation with magnetic resonance imaging volume estimation and surgical specimen weight in patients with benign prostatic hyperplasia. J Clin Ultrasound. 1996;24(4):169 174. 10.1002/(SICI)1097-0096(199605)24:4<169::AID-JCU2>3.0.CO;2-D) 8727414

[13] Martins T Mussi TC Baroni RH . Prostate volume measurement by multiparametric magnetic resonance and transrectal ultrasound: comparison with surgical specimen weight. Einstein (Sao Paulo). 2020;18:eAO4662. 10.31744/einstein_journal/2020AO4662) PMC6986883 32022105

[14] Bazinet M Karakiewicz PI Aprikian AG et al. Reassessment of nonplanimetric transrectal ultrasound prostate volume estimates. Urology. 1996;47(6):857 862. 10.1016/S0090-4295(96)00068-4) 8677577

[15] Baltaci S Yagci C Aksoy H Elan AH Gögüs O . Determination of transition zone volume by transrectal ultrasound in patients with clinically benign prostatic hyperplasia: agreement with enucleated prostate adenoma weight. J Urol. 2000;164(1):72 75. 10.1016/S0022-5347(05)67452-6) 10841600

[16] Wilson LC Gilling PJ Williams A et al. A randomised trial comparing holmium laser enucleation versus transurethral resection in the treatment of prostates larger than 40 grams: results at 2 years. Eur Urol. 2006;50(3):569 573. 10.1016/j.eururo.2006.04.002) 16704894

[17] Abt D Hechelhammer L Müllhaupt G et al. Comparison of prostatic artery embolisation (PAE) versus transurethral resection of the prostate (TURP) for benign prostatic hyperplasia: randomised, open label, non-inferiority trial. BMJ. 2018;361:k2338. 10.1136/bmj.k2338) 29921613 PMC6006990

[18] Gilling PJ Wilson LC King CJ Westenberg AM Frampton CM Fraundorfer MR . Long-term results of a randomized trial comparing holmium laser enucleation of the prostate and transurethral resection of the prostate: results at 7 years. BJU Int. 2012;109(3):408 411. 10.1111/j.1464-410X.2011.10359.x) 21883820

[19] Meikle AW Stephenson RA Lewis CM Middleton RG . Effects of age and sex hormones on transition and peripheral zone volumes of prostate and benign prostatic hyperplasia in twins. J Clin Endocrinol Metab. 1997;82(2):571 575. 10.1210/jcem.82.2.3720) 9024256

[20] Lee JS Chung BH . Transrectal ultrasound versus magnetic resonance imaging in the estimation of prostate volume as compared with radical prostatectomy specimens. Urol Int. 2007;78(4):323 327. 10.1159/000100836) 17495490

[21] Rahmouni A Yang A Tempany CM et al. Accuracy of in-vivo assessment of prostatic volume by MRI and transrectal ultrasonography. J Comput Assist Tomogr. 1992;16(6):935 940. 10.1097/00004728-199211000-00020) 1385499

[22] Jeong CW Park HK Hong SK Byun SS Lee HJ Lee SE . Comparison of prostate volume measured by transrectal ultrasonography and MRI with the actual prostate volume measured after radical prostatectomy. Urol Int. 2008;81(2):179 185. 10.1159/000144057) 18758216

[23] Aprikian S Luz M Brimo F et al. Improving ultrasound-based prostate volume estimation. BMC Urol. 2019;19(1):68. 10.1186/s12894-019-0492-2) PMC6657110 31340802

[24] Rodriguez E Jr Skarecky D Narula N Ahlering TE . Prostate volume estimation using the ellipsoid formula consistently underestimates actual gland size. J Urol. 2008;179(2):501 503. 10.1016/j.juro.2007.09.083) 18076916

[25] MacMahon PJ Kennedy AM Murphy DT Maher M McNicholas MM . Modified prostate volume algorithm improves transrectal US volume estimation in men presenting for prostate brachytherapy. Radiology. 2009;250(1):273 280. 10.1148/radiol.2501080290) 19092098

